# Estimating demographic parameters from large-scale population genomic data using Approximate Bayesian Computation

**DOI:** 10.1186/1471-2156-13-22

**Published:** 2012-03-27

**Authors:** Sen Li, Mattias Jakobsson

**Affiliations:** 1 Department of Evolutionary Biology, EBC, Uppsala University, Norbyvägen 18D, Uppsala SE-75236, Sweden; 2 Science for Life Laboratory, Uppsala University, Uppsala, Sweden

## Abstract

**Background:**

The Approximate Bayesian Computation (ABC) approach has been used to infer demographic parameters for numerous species, including humans. However, most applications of ABC still use limited amounts of data, from a small number of loci, compared to the large amount of genome-wide population-genetic data which have become available in the last few years.

**Results:**

We evaluated the performance of the ABC approach for three 'population divergence' models - similar to the 'isolation with migration' model - when the data consists of several hundred thousand SNPs typed for multiple individuals by simulating data from known demographic models. The ABC approach was used to infer demographic parameters of interest and we compared the inferred values to the true parameter values that was used to generate hypothetical "observed" data. For all three case models, the ABC approach inferred most demographic parameters quite well with narrow credible intervals, for example, population divergence times and past population sizes, but some parameters were more difficult to infer, such as population sizes at present and migration rates. We compared the ability of different summary statistics to infer demographic parameters, including haplotype and LD based statistics, and found that the accuracy of the parameter estimates can be improved by combining summary statistics that capture different parts of information in the data. Furthermore, our results suggest that poor choices of prior distributions can in some circumstances be detected using ABC. Finally, increasing the amount of data beyond some hundred loci will substantially improve the accuracy of many parameter estimates using ABC.

**Conclusions:**

We conclude that the ABC approach can accommodate realistic genome-wide population genetic data, which may be difficult to analyze with full likelihood approaches, and that the ABC can provide accurate and precise inference of demographic parameters from these data, suggesting that the ABC approach will be a useful tool for analyzing large genome-wide datasets.

## Background

In evolutionary biology and population genetics, several approaches for inferring demographic or genetic parameters are based on Bayesian statistical inference [1-3]. Bayesian statistics is a general framework based on Bayes' theorem that can be used to estimate unknown parameters. Bayesian inference uses the following relationship

P(θ|D)∝P(θ)⋅P(D|θ),

where *P*(*θ|D*) is the conditional distribution of some parameter of interest (*θ*) given the data (*D*), *P*(*θ*) is the prior distribution of the parameter, and *P*(*D|θ*) is the probability of the data given the parameter (the likelihood function: *L*(*θ*) = *P*(*D|θ*). According to the expression above, the conditional distribution *P*(*θ|D*) of the parameter given the data, which is called the posterior distribution, is proportional to the prior distribution and the likelihood. For most practical cases in evolutionary biology and population genetics, the likelihood function is very difficult to compute because of the large amount of data and the potentially complex models, and exact approaches requiring evaluation of the full likelihood are often restricted to simple evolutionary models [4].

Approximate Bayesian Computation (ABC) can be used to make inference for complex models with high dimensional data [5]. First, in order to overcome the difficulty in evaluating exact likelihoods, ABC approximates the likelihoods of the parameters based on a tolerance level (with respect to some metric) for the difference between observed and simulated data. As the tolerance level goes to zero, ABC produces a sample from the posterior distribution. Second, to overcome the difficulty in evaluating high dimensional data, ABC evaluates summary statistics that reduce the dimensionality of the data. The summary statistics are ideally chosen so that they capture as much information as possible from the data about the parameter(s) of interest. However, because only non-sufficient summary statistics exist for most complex models and parameters of interest, the effects of such statistics will be case-dependent, and the effect of mapping the data space to arbitrarily chosen non-sufficient summary statistics space is not well-known. Tavaré *et al. *(1997) [5] described a straightforward rejection-algorithm for approximate Bayesian inference, which was extended by Pritchard *et al. *(1999) [6] to allow some level of deviation between the observed and simulated data. In short, their algorithm proceeds as follow: Simulate a large number of datasets based on different values for some parameter of interest, where the parameter values are sampled from a prior distribution. Next, calculate summary statistics of simulated datasets, accept or reject parameter values on the basis of the difference between simulated summary statistics and the summary statistics of some observed data. Finally, the accepted parameter values represent an approximate sample from the posterior distribution of the parameter of interest. There are several more advanced versions of the basic rejection approach, such as local linear regression adjustment [7], non-linear feed forward neural networks [8], ABC with Markov chain Monte Carlo [3], ABC with sequential Monte Carlo [9].

Population divergence models, or 'isolation with migration' models, have been used extensively in order to describe properties of populations and species, and to explore increasingly complex demographic scenarios e.g. [10-18]. These models can often be good approximations of scenarios that involve populations splitting off from an ancestral population e.g. [13,18], such as the colonization of islands or distant continents, or the domestication of livestock and crops. In recent years, ABC has also been used to infer demographic parameters of humans from genetic data. For example, Fagundes *et al. *(2007) [19] estimated several demographic and historical parameters using divergence models, such as the timing of modern humans' exodus from Africa and the time of colonization of the Americas, based on data from 50 nuclear loci sequenced in African, Asian and Native American samples. Similarly, Wegmann *et al. *(2009) [20] applied ABC with a Markov chain Monte Carlo approach to estimate divergence times and migration rates between three African populations based on 331 microsatellites. Bertorelle *et al. *(2010) [21] conducted a survey about ABC related publications since 2002. In their survey, they found that 43% publications used microsatellite markers as the source of genetic information, which was the most common source, and the remaining fraction was divided between nuclear and mitochondrial sequence data. The median value of the number of loci for STR and nuclear sequence data was 9 and 19, respectively. Bertorelle *et al. *(2010) [21] concluded that most applications of ABC still use limited amounts of data, often due to using a small number of loci, compared to the amount of genome-wide population-genetic data which has become available in the last few years [22-26]. Recently, Wollstein *et al. *(2010) [27] used an ABC approach to investigate the demographic history of Oceania based on approximately 1 million SNPs. Based on that data, and accounting for ascertainment bias, they could provide a more detailed picture of human history and the peopling of Oceania than has previously been painted. However, most studies that use ABC are based on a small number of markers (e.g. Bertorelle *et al. *(2010) [21]) leading to, in many cases, imprecise parameter estimation [28], and questions about of the power of ABC under some scenarios [29,30].

In this article, we investigate the performance and power of the ABC approach when we have access to large amounts of genome-wide population-genetic data. We study the ABC approach with local linear regression adjustment for several population divergence models. Simulated data is generated under 'human-like' conditions and from a particular known demographic model (some 150,000 to 300,000 SNPs are generated). Three population divergence models with increasing complexity are investigated, and we compare estimation accuracy of particular parameters under the different models. The effect of the number of loci on the performance of the ABC approach is also investigated.

## Methods

### Population models

**Figure 1:**
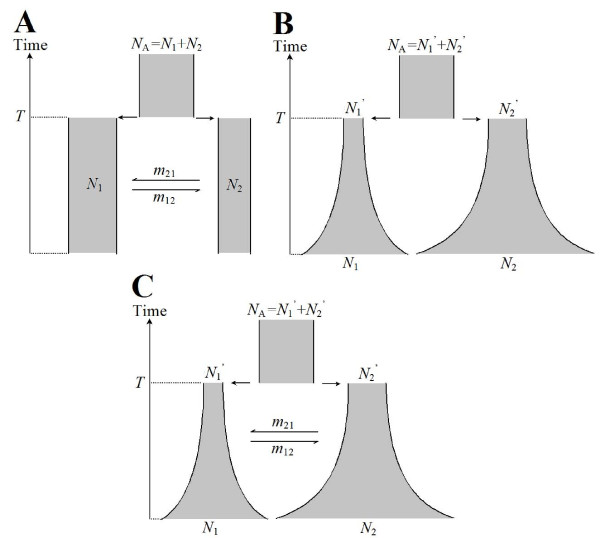
**Population models**. A) Model 1: a simple divergence model, with two sub-populations that have constant sizes (*N*_1 _and *N*_2_). Migration occur after divergence event (at time *T*) with rate *m*_12 _and *m*_21_; B), model 2: a divergence model with exponential growth. After the divergence time *T*, two sub-populations (of size *N*_1_^' ^and *N*_2_^'^) grow with exponential rates *α*_1 _and *α*_2_, and the population sizes at present are *N*_1 _and *N*_2_. There is no migration between the sub-populations; C) model 3: Composite model of model 1 and model 2. The same as model 2, but with migration occurring at rate *m*_12 _and *m*_21 _after divergence time.

We investigate three different population divergence models. These models were chosen to be similar to commonly studied population models, such as the 'isolation with migration' model, and to represent an increasing complexity. In the first model (Figure 1A), an ancestral population with size *N*_A _was split into two sub-populations (population 1 and population 2) at time *T *before present (scaled by 4*N_e _*generations, where *N_e _*= 10,000). Sub-populations had a constant size of *N*_1 _= *N_e _*and *N*_2 _= 0.5*N_e_*, respectively (where *N*_A _= *N*_1 _+ *N*_2_). Migration between the two sub-populations occurred at rate *m*_12 _(from population 1 to population 2) and *m*_21 _(from population 2 to population 1), where the migration rate *m *= 4*N_e_M *and *M *is the fraction of migrants per generation. In this model, we treated the parameters *T*, *m*_12 _and *m*_21 _as unknown and we attempted to infer their values based on simulated genetic data. The sub-population sizes *N*_1 _and *N*_2 _were assumed to be known for this case.

In the second model (Figure 1B), an ancestral population was split into two sub-populations with size *N*_1_^' ^and *N*_2_^' ^at time *T*. The size of the ancestral population was set to *N*_A _= *N*_1_^' ^+ *N*_2_^'^. Each sub-population grew exponentially starting at time *T *with different rates *α*_1 _and *α*_2_. At present, the size of each sub-population was *N*_1 _and *N*_2_. In this model, we assumed that there was no migration between populations. We aim to estimate past population sizes *N*_1_^' ^and *N*_2_^'^, and present population sizes *N*_1 _and *N*_2_. Since both past and present population sizes will be drawn from prior distributions for each proposed set of parameters, the growth rates *α*_1 _and *α*_2 _are fixed and can be computed by $\alpha_{i}=\text{ln}\left(N_{i}/N_{i}^{\prime }\right),i=1\;or\;2$. The divergence time *T *was assumed to be known in this model (T = 0.1 × 4*N_e _*= 4,000 generations).

The third model (Figure 1C) was a combination of model 1 and model 2, we treated all parameters as unknown, and we were interested in estimating all these seven parameters: divergence time *T*, migration rates *m*_12 _and *m*_21_, past population sizes *N*_1_^' ^and*N*_2_^'^, and present population sizes *N*_1 _and *N*_2_.

### Simulated data

Population-genetic SNP data, comparable in size to human SNP-chip data or large scale re-sequencing data, was simulated using Hudson's *ms *program [31]. We simulated 10,000 genome-regions each of size 100 kb, for a total of 200 chromosomes (200 haploid individuals or 100 diploid individuals), 100 chromosomes from each sub-population. The mutation rate (*θ *= 4*N*_e_*μ*) per genome-region was set to 5, the recombination rate (*ρ = *4*N*_e_*r*) per genome-region was set to 40. By assuming an effective population size of *N*_e _= 10,000, the mutation rate *θ *corresponded to *μ*_s _= 1.25 × 10^-9 ^per base pair per generation, and the recombination rate *ρ *corresponded to *r*_s _≈ 1.00 × 10^-8 ^per base pair per generation. The recombination rate *r *was chosen to match recombination rates estimated from large-scale genomic data and the mutation rate *μ *was chosen to correspond to an incomplete set of SNPs (which was lower than the value ~10^-8 ^for human genome) [32], on the order of 1/8^th ^of all SNPs in the region. Furthermore, SNPs with minor allele frequency less than or equal to 5% were removed in order to mimic ascertained SNPs. With these values of the parameters, one replicate of the simulated data resulted in a few hundred thousand SNPs. The 10,000 genome-regions were assumed to be independent of each other. The *ms *commands for generating data under the three models are provided in the Additional file 1.

### ABC with local linear regression adjustment

To estimate demographic parameters, we used the ABC approach with local linear regression adjustment, introduced by Beaumont *et al. *(2002) [7] that also utilize smooth weighting for candidate parameters instead of only using a rejection algorithm (e.g. Pritchard *et al. *1999) [6]. The linear regression is an innovation that has been successful in reducing the computational load of ABC, but it can produce nonsense values (due to post-processing of sampled values) for the posterior distribution that fall outside the prior distribution. Parameter values not included in the prior distribution cannot appear in the posterior distribution in parametric models as a consequence of Bayes' theorem, and a transformation of accepted parameters vales [33] solves this issue so that only values that appear in the prior distribution can appear in the posterior distribution.

In detail, our ABC procedure can be described as follows:

(1) Sample a set of candidate parameters, *θ_i_*, from each of the prior distributions;

(2) Simulate population-genetic data using the sampled parameter-values *θ_i _*under a particular model;

(3) Compute summary statistics from simulated data;

(4) Compute Euclidean norm for the differences between the set of simulated summary statistics, $S_{i}^{*}\equiv \left(S_{i1}^{*},\ldots ,S_{iq}^{*}\right),$ and the set of observed summary statistics, $S\equiv \left(S_{1},\ldots ,S_{q}\right)$, so that $∥S_{i}^{*}-S∥=\sqrt{\sum_{j=1}^{q}{\left(S_{ij}^{*}-S_{j}\right)}^{2}}$, where *q *is the number of summary statistics. All summary statistics were standardized before computing the Euclidean norm.

(5) Select a fixed fraction of candidate parameter-sets that have the smallest values of $∥S_{i}^{*}-S∥$ and use the Epanechnikov kernel to weight the candidate parameter-sets. Adjust candidate parameters by using a local linear regression approach [7].

We generated 50,000 replicate simulated dataset for each model and choice of prior that were investigated, which corresponds to 500 million simulated genome-regions of size 100 kb. The tolerance level was set to 1% (except for the investigation of tolerance level). In order to assure that the estimated posterior distribution obtained by the local linear regression approach stayed within the bounds of the prior distribution, we transformed the values of the accepted parameters in the rejection step before the regression step [33]. The obtained adjusted parameter-values are draws from the posterior distributions, and can be used as an approximation of the posterior distributions of the parameters of interest.

### Summary statistics

We used eight different classes of summary statistics for the ABC approach. All summary statistics were computed individually for the two sub-populations, except for *F_ST_*, resulting in a total of 15 summary statistics. Many of the summary statistics were based on 'haplotypes' and the genetic data were assumed to be phased. We define a haplotype locus by the chunk of DNA that extends from the SNP position *a *along the genome to the SNP position *a + w *(for a particular window size *w*). A haplotype-allele is defined as the combination of variants at all SNPs within the window *w *for a particular chromosome (and for a particular haplotype locus) [34]. The 8 different classes of summary statistics are:

(1) Haplotype heterozygosity (HHA) [34] of the entire genome-region (the window extended over the entire 100 kb genome-region). The statistic was computed from the frequency _100_*h_i _*of each haplotype-allele *i *in a particular population,

$$HHA=\frac{n}{n-1}\left(1-{\sum {}_{i}}_{100}h_{1}^{2}\right),$$

where *n *is the number of sampled chromosomes.

(2) Average heterozygosity of 10 kb-haplotype-windows (HAW). For a window of size 10 kb, the haplotype heterozygosity in a particular window *j *was computed as

$$H_{j}=1-{\sum {}_{i}}_{10}h_{ij}^{2},$$

where _10_*h_ij _*is the frequency of the haplotype-allele *i *in the 10 kb window *j*. The window moves one SNP at the time, and the average haplotype heterozygosity of all windows is

$$HAW=\frac{n\sum_{j}H_{j}}{\left(n-1\right)S},$$

where *n *is the number of sampled chromosomes, and *S *is the number of SNPs.

(3) The average heterozygosity of all segregating sites (HSS). The *HSS *statistic was computed from

$$HSS=\frac{n\sum_{j}\left(1-\sum_{i}f_{ij}^{2}\right)}{\left(n-1\right)S},$$

where *f_ij _*is the frequency of allele *i *for SNP *j*, *n *is the number of sampled chromosomes, and *S *is the number of SNPs.

(4) Linkage disequilibrium (LDR), measured as *r*^2 ^[35]. For each pair of SNPs that were between 9 and 11 kb apart, *r*^2 ^was computed using

$$r^{2}=\frac{{\left(x_{11}-j_{1}k_{1}\right)}^{2}}{j_{1}j_{2}k_{1}k_{2}},$$

where *j*_1 _and *j*_2 _denote the frequency of allele 1 and allele 2 at SNP J and *k*_1 _and *k*_2 _denote the frequency of allele 1 and allele 2 at SNP K, and *x*_11 _denotes the frequency of the J_1_K_1 _haplotype. The average *r*^2 ^is computed across all pairs of SNPs (that are located between 9 kb and 11 kb from each other) to get LDR.

(5) The number of distinct haplotype-alleles (NOA) per genome-region.

(6) The number of private haplotype-alleles in each sub-population (NPA) per genome-region.

(7) Tajima's D (TAD). Computed for all SNPs in each genome-region following [36].

(8) *F_ST _*(FST). Computed for all SNPs in each genome-region using equation 5.3 in [37].

The summary statistics were computed for each of the two populations, except for *F_ST_*, and all summary statistics were averaged across the 10,000 genome regions. We also tested to use the variances (across the genome regions) instead of the means in the ABC procedure, see discussion.

## Results

### Comparison of population models

To generate simulated datasets that represent potentially empirical datasets, we fixed a particular true value of each parameter (Table 1) and simulated "observed" datasets from the particular model under consideration. We used these "observed" datasets to evaluate how well the ABC approach could recover the true parameter values. By this setup, we could compare the true parameter values to the inferred parameter values and evaluate the performance of the ABC approach under different conditions. The prior distributions of each parameter in the ABC framework are given in Table 1. Note that we scaled the values of *T*, *m*_12_, and *m*_21 _by 4*N*_e_, where *N*_e _was set to 10,000. We start by investigating single "observed" datasets to mimic the conditions of empirical studies, followed by investigating multiple "observed" datasets. An outline of the various investigations is given in Additional file 1: Table S1.

**Table 1:** True values and prior distributions of each parameter of the 3 models

Model		Parameter	True value	Prior distribution (uniform)
		*T*	0.1	(0, 0.5)
	model 1	*m*_12_	2	(0, 5)
		*m*_21_	1	(0, 5)
	
model 3		*N*_1_^'^	2,000	[100, 10,000]
	model 2	*N*_2_^'^	5,000	[100, 10,000]
		*N*_1_	100,000	[10,000, 200,000]
		*N*_2_	150,000	[10,000, 200,000]

**Figure 2:**
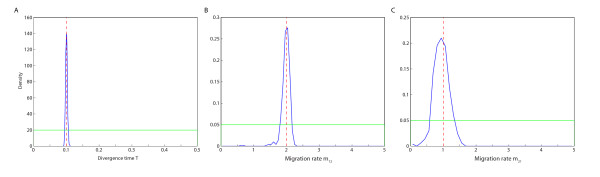
**Estimated posterior distribution of each parameter in model 1**. A) Divergence time *T*, B) migration rate from population 1 to population 2 (*m*_12_), and C) migration rate from population 2 to population 1 (*m*_21_). The vertical dashed red line indicates the true value of the parameter, the estimated posterior distribution of parameter is shown as a blue line, and the prior distribution of the parameter is shown in green.

For the simple isolation with migration model 1, the parameter estimation turned out to be quite accurate. Table 2 gives the means and the 95% credible intervals of the posterior samples of *T*, *m*_12 _and *m*_21_, and Figure 2 shows the prior and the estimated posterior distributions for the same parameters. For the divergence time *T*, the mean of the posterior sample was 0.30% smaller than the true value, and the 95% credible interval was narrow. For the migration rate *m*_21_, the mean of the posterior sample was 5.46% smaller compared to a true value of 1 (one) and the 95% credible interval was [0.5033, 1.3935]. The estimate of the migration rate *m*_12 _turned out to be very close to the true value of *m*_12 _(only 0.99% greater than the true value), but its 95% credible interval was about as wide as for *m*_21_.

**Table 2:** Summary of the posterior sample for each parameter in model 1

Parameter	True value	Mean	Difference	95% interval
*T*	0.1	0.1003	0.30%	[0.0952, 0.1063]
*m*_12_	2	1.9803	0.99%	[1.6516, 2.1560]
*m*_21_	1	0.9454	5.46%	[0.5033, 1.3935]

The true value, the mean of the posterior sample, the difference of the estimated mean value and the true value, and the 95% credible interval of the posterior sample are given for each parameter

**Figure 3:**
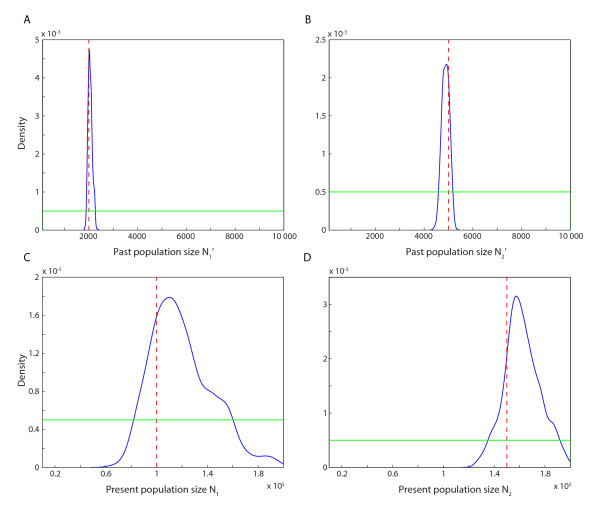
**Estimated posterior distribution of each parameter in model 2**. A) Past population size *N*_1_^'^, B) past population size *N*_2_^'^, C) present population size *N*_1_, and D) present population size *N*_2_. The vertical dashed red line indicates the true value of each parameter, the blue line shows the estimated posterior distribution of each parameter, and the green line shows the prior distribution of each parameter.

For model 2, the means of the posterior samples of past population sizes were slightly closer to the true values than the means for the present population sizes, but the 95% credible intervals of *N*_1_^' ^and *N*_2_^' ^were much more narrow compared to 95% credible intervals of *N*_1 _and *N*_2_, indicating that present population sizes are more difficult to estimate. Table 3 shows the mean and the 95% credible interval of the posterior samples of the past population sizes (*N*_1_^' ^and *N*_2_^'^), and the present population sizes (*N*_1 _and *N*_2_). Figure 3 shows the prior and the estimated posterior distributions for the same parameters.

**Table 3:** Summary of the posterior sample for each parameter in model 2

Parameter	True value	Mean	Difference	95% interval
*N*_1_^'^	2,000	2,054	2.70%	[1,907, 2,244]
*N*_2_^'^	5,000	4,883	2.34%	[4,580, 5,141]
*N*_1_	100,000	119,913	19.91%	[82,623, 177,468]
*N*_2_	150,000	162,167	8.11%	[135,291, 190,948]

The true value, the mean of the posterior sample, the difference of the estimated mean value and the true value, and the 95% credible interval of the posterior sample are given for each parameter

**Figure 4:**
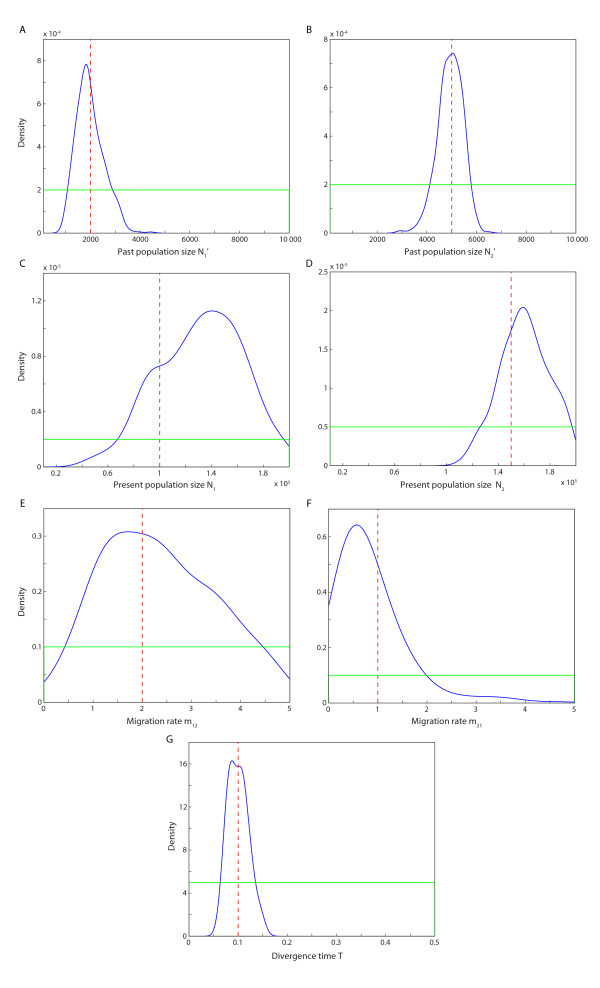
**Estimated posterior distribution of each parameter in model 3**. A) Past population size *N*_1_^'^, B) past population size *N*_2_^'^, C) present population size *N*_1_, D) present population size *N*_2_, E) migration rate from population 1 to population 2 (*m*_12_), F) migration rate from population 2 to population 1 (*m*_21_), G) divergence time *T*. The vertical dashed red line indicates the true value of each parameter, the blue line shows the estimated posterior distribution of each parameter, and the green line shows the prior distribution of each parameter.

Model 3 - a combination of model 1 and model 2 - is more complex, but also more flexible than models 1 and 2. For model 3, we estimated seven parameters compared to the three and the four parameters in models 1 and 2, respectively. A summary of the posterior samples for the parameters in model 3 is given in Table 4 and the estimated posterior distributions are shown in Figure 4. For model 3, the divergence time and the two past population sizes were estimated quite well; the mean values of the posterior samples were close to true values and the 95% credible intervals were fairly narrow. The means of the posterior samples of the two migration rates were relatively far from the true values (17.27% and 16.44%) and the 95% credible intervals were also wide (see Table 4). The two present population sizes were somewhat poorly estimated (Table 4).

**Table 4:** Summary of the posterior sample for each parameter in model 3

Parameter	True value	Mean	Difference	95% interval
*T*	0.1	0.1000	0.00%	[0.0654, 0.1449]
*m*_12_	2	2.3454	17.27%	[0.4864, 4.6692]
*m*_21_	1	0.8356	16.44%	[0.0686, 3.1288]
*N*_1_^'^	2,000	1,987	0.65%	[1,106, 3,178]
*N*_2_^'^	5,000	4,943	1.14%	[3,838, 5,828]
*N*_1_	100,000	131,615	31.62%	[66,355, 192,624]
*N*_2_	150,000	160,328	6.89%	[122,769, 194,806]

The true value, the mean of the posterior sample, the difference of the estimated mean value and the true value, and the 95% credible interval of the posterior sample are given for each parameter

We compared the three parameters in common between model 1 and model 3, and the four parameters in common between model 2 and model 3. Generally, the estimation of each parameter was more accurate under model 1 or model 2 compared to model 3; both the mean values and the 95% credible intervals of the posterior samples were more precise for model 1 and 2 compared to model 3. Especially the 95% credible intervals estimated in models 1 and 2 were much smaller compared to the 95% credible intervals in model 3. These observations were not surprising and reflect the notion that the more complex a model is, the less accurate will the parameter estimation be (given the same estimation conditions). However, the means of the posterior samples of *T*, *N*_1_^'^, *N*_2_^'^, were still quite close to the true values even for the complex model 3, but the migration rates and present population sizes were more difficult to estimate as illustrated by the wide credible intervals (Figure 4).

**Figure 5:**
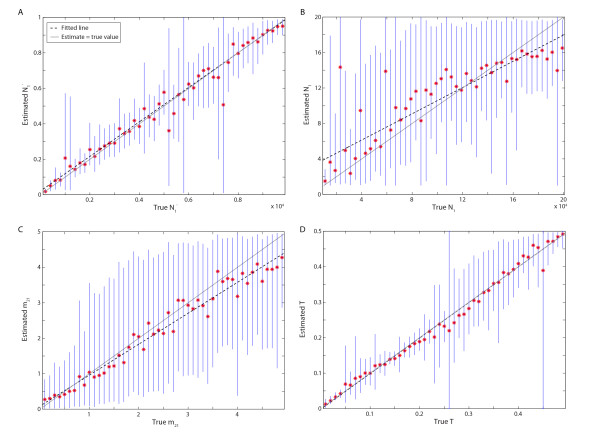
**Performance of the ABC with local linear regression, for estimating past and present population sizes, migration rate, and divergence time**. The red stars indicate the means of the posterior sample and blue lines give the 95% credible intervals. The dashed black line shows the linear fit to the means and the gray solid line shows the ideal case, when the estimate equals the true value.

We generated 195 "observed" datasets for model 3 where the true past population size *N*_1_' ranged from 200 to 9,800, the true current population size *N*_1 _ranged from 11,000 to 198,000, the true migration rate *m*_21 _ranged from 0.1 to 4.9, and the true divergence time *T *ranged from 0.01 to 0.49 (the other parameters were set to the same values as above, see Table 4). These datasets were generated to investigate to what extent the observations from single "observed" datasets generalize to a wide range of true values for various parameters and multiple instances of estimating parameters using ABC. In most of cases, the ABC with local linear regression adjustment estimated all four parameters satisfactory (Figure 5), including the difficult current population size and the migration rate, albeit that the credible intervals were large. For the past population size and the divergence time, there were a few exceptional cases where the 95% credible intervals extended over almost the entire range of the prior (e.g. *T *= 0.26 and *N*_1_' = 5,800). For these cases, the set of summary statistics for accepted parameter-values included one or more extreme outlier, which in turn caused the local linear regression to produce a wide range of adjusted parameter-values since the normal least-square estimation for the regression model is non-robust to outliers [38].

**Figure 6:**
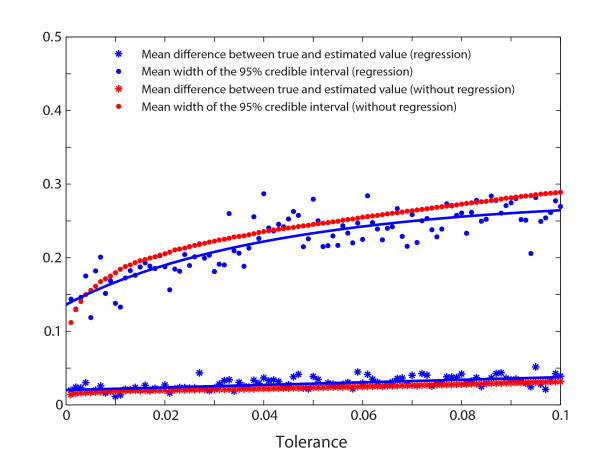
**Influence of the tolerance level**. The mean (across 49 choices of *T*) difference between the true and the estimated divergence time *T *as a function of the tolerance level (blue stars for using regression and red stars for using rejection only). The mean (across 49 choices of *T*) width of the 95% credible interval for the estimated *T *as a function of the tolerance level (blue filled circles for using regression and red filled circles for using rejection only). For comparison, fitted lines are included for the results of ABC with local linear regression.

We also investigated a range of tolerance levels to determine its impact on the accuracy of the parameter estimation. For each of the 49 "observed" datasets where the true *T *ranged from 0.01 to 0.49, we varied the tolerance level from 0.2% to 10%. For each tolerance level, the mean (across the 49 choices of the true value of *T*) difference between the true and the estimated *T *(mean of the posterior sample) was computed (Figure 6). The difference between the true and the estimated *T *decreased as the tolerance decreased (Pearson correlation: 0.61, p < 10^-10^). Furthermore, the width of the 95% credible region also decreased with decreasing tolerance levels (Pearson correlation: 0.82, p < 10^-24^, Figure 6). For comparison, if the use a standard rejection algorithm instead of ABC with local linear regression, the difference between the true and the estimated *T *turned out to be very similar, but the width of the 95% credible region was slightly smaller when using local linear regression. Hence, as long as the number of accepted replicate simulations was reasonable - in this case a hundred or greater - the parameter estimation using ABC benefits from a low tolerance levels.

Both mutation rates and recombination rates can vary across the genome, and in most practical ABC analyses the empirical data will come from various genome regions with potentially different underlying mutation and recombination rates. In order to explore the effect of using fixed mutation and recombination rates in the ABC procedure, we simulated "observed" datasets where the mutation rate or the recombination rate of each genome region was randomly drawn from a normal distribution [norm(*θ*, 0.2) or norm(*ρ*, 1)]. The ABC procedure uses fixed values for the mutation and the recombination rates (*θ *= 5 and *ρ *= 40). In the case that the mutation rate or the recombination rate of the ABC was similar to the mean of the distribution of the mutation rate or the recombination rate, the ABC estimates were not affected much (Table 5) compared to if the mutation and recombination rates were fixed for the "observed" data (Table 4). However, if the simulations in the ABC procedure used a mutation rate that was a third of the mean of the "observed" data or three times larger than the mean of the "observed" data, the ABC estimates were not very accurate (Table 5). If the simulations in the ABC procedure used a recombination rate that was half of the mean of the "observed" data the ABC estimates were also inaccurate, but if the ABC procedure used a recombination rate that was twice as large as the mean of the "observed" data, the ABC estimates were less affected and stayed reasonably accurate (Table 5).

**Table 5:** The estimation of divergence time, migration rates, past population sizes and present population sizes in model 3 when the mutation rate or the recombination rate varies across the genome regions

Parameter (true value)		Varying mutation rate (*θ *= 4*N_e_μ*)			Varying recombination rate (*ρ *= 4*N_e_r*)		
		*E*[*θ*] = 5/3	*E*[*θ*] = 5	*E*[*θ*] = 5 × 3	*E*[*ρ*] = 40/2	*E*[*ρ*] = 40	*E*[*ρ*] = 40 × 2
***T *(0.1)**	**Mean**	0.0700	0.1070	0.1168	0.0662	0.1154	0.1489
	**95% CI**	[0.0252, 0.1383]	[0.0656, 0.1759]	[0.0726, 0.1864]	[0.0274, 0.1259]	[0.0626, 0.1883]	[0.1018, 0.2054]
***m*_12 _(2.0)**	**Mean**	3.1771	2.4135	4.9080	1.5306	2.5406	2.6228
	**95% CI**	[0.4462, 4.8543]	[0.4741, 4.6774]	[4.5929, 4.9939]	[0.0379, 4.2540]	[0.5208, 4.6828]	[1.0433, 4.6559]
***m*_21 _(1.0)**	**Mean**	0.7207	0.9144	0.0476	4.6749	0.9631	0.1092
	**95% CI**	[0.0465, 3.4811]	[0.1033, 3.0788]	[0.0000, 0.2378]	[2.8810, 4.9834]	[0.1032, 3.1232]	[0.0190, 0.3659]
***N*_1_^' ^(2,000)**	**Mean**	1,268	2,349	9,014	1,820	2,474	4,779
	**95% CI**	[499, 2,460]	[1195, 4,227]	[7,253, 9,884]	[796, 3,402]	[1,186, 4,390]	[2,738, 6,790]
***N*_2_^' ^(5,000)**	**Mean**	1,777	4,777	9,338	3,562	4,888	4,104
	**95% CI**	[770, 3,361]	[3,344, 6,235]	[7,878, 9,971]	[2,301, 4,836]	[3,324, 6,276]	[2,620, 5,496]
***N*_1 _(100,000)**	**Mean**	164,116	116,700	75,603	44,265	115,277	192,358
	**95% CI**	[94,583, 198,448]	[47,239, 191,310]	[40,493, 133,925]	[13,558, 159,882]	[44,780, 191,187]	[177,723, 199,615]
***N*_2 _(150,000)**	**Mean**	183,527	154,968	24,650	45,778	151,010	196,899
	**95% CI**	[14,273, 199,210]	[102,065, 195,442]	[12,964, 75,434]	[16,968, 140,253]	[98,170, 195,246]	[190,142, 199,659]

For each "observed" dataset, we randomly draw a mutation rate or a recombination rate for each genome region from a normal distribution with parameter (*E*[*θ*], 0.2) or (*E*[*ρ*], 1). The ABC procedure uses fixed values for the mutation and the recombination rates, *θ *= 5 and *ρ *= 40. The means and 95% CIs are averaged over 50 replicate "observed" datasets. Compare with Table 4 which shows the ABC estimates when the "observed" data are generated from a model with fixed values for the mutation and the recombination rates

### Choosing a poor prior

In order to evaluate how a poor choice of prior affects the inference based on the ABC approach with local linear regression adjustment, we set a number of true values of the divergence time *T *outside the prior distribution of (0, 0.5) for model 3. All other parameter settings of the model and the priors were the same as in Table 1. We repeated the ABC procedure to infer the parameter *T *for this case where the prior distribution does not cover the true parameter values. The posterior sample was limited to the range of the prior distribution and the mean of the posterior sample for each case hit the upper bound of the prior distribution (Table 6). For comparison, if the ABC procedure with local linear regression was implemented without the transformation step, the mean of the posterior samples extended outside the upper bound of the prior, and the 95% credible interval extended well beyond the bounds on the prior distribution (but note that such results violate Bayes' theorem; Table 6). These observations were indications that some model assumption was violated, such as choosing a prior distribution that does not cover the true parameter value. By changing the prior of *T *to (0.3, 0.8) for the case of the true *T *= 0.7, and repeating the ABC analysis (10,000 replicate simulations), the mean of the posterior sample (0.7274) was quite close to the true value, and the 95% interval [0.6350, 0.7946] was fairly narrow around the true value.

**Table 6:** Estimation of divergence time *T *for model 3 in cases where the prior distribution does not encompass the true parameter value

True *T*	With transformation		Without transformation		Without regression	
	
	Mean	95% interval	Mean	95% interval	Mean	95% interval
0.60	0.4994	[0.4954, 0.5000]	0.6226	[0.0977, 1.1443]	0.4607	[0.3945, 0.4991]
0.65	0.5000	[0.5000, 0.5000]	0.5952	[0.5618, 0.6297]	0.4633	[0.4039, 0.4991]
0.70	0.5000	[0.5000, 0.5000]	0.7502	[0.5228, 0.9915]	0.4669	[0.4158, 0.4994]
0.75	0.5000	[0.5000, 0.5000]	0.5447	[0.4450, 0.6692]	0.4703	[0.4216, 0.4995]
0.80	0.5000	[0.5000, 0.5000]	1.2404	[0.4929, 1.8773]	0.4729	[0.4275, 0.4994]
0.85	0.5000	[0.5000, 0.5000]	0.7836	[0.5244, 0.9533]	0.4731	[0.4308, 0.4994]
0.90	0.5000	[0.5000, 0.5000]	0.6255	[0.5325, 0.7240]	0.4738	[0.4312, 0.4994]
0.95	0.5000	[0.5000, 0.5000]	0.6322	[0.4716, 0.7902]	0.4749	[0.4376, 0.4994]
1.00	0.5000	[0.5000, 0.5000]	0.6887	[0.5749, 0.8131]	0.4754	[0.4358, 0.4991]

The means and the 95% credible intervals of the posterior samples are given for the ABC estimate using regression and transformation, using regression but without transformation, and without using regression (i.e., only using rejection). See text for a detailed explanation of the different versions of the ABC approach

### Comparison of summary statistics

**Figure 7:**
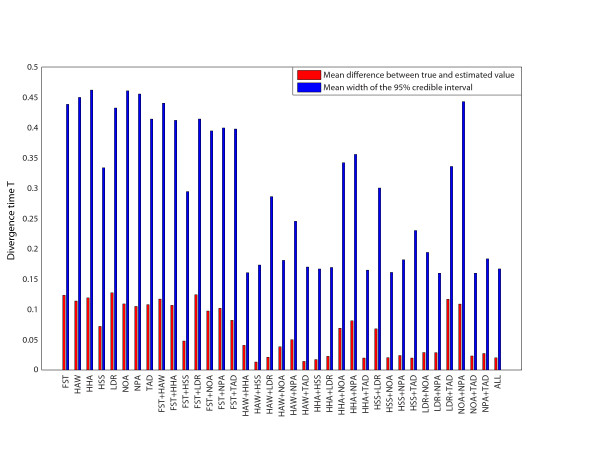
**The mean (across 49 choices of true *T*) difference between the true and estimated divergence time *T *(red) and the mean width of the 95% credible interval of the posterior sample (blue) given by single summary statistics, pairs of summary statistics, and the combination of all eight summary statistics**. The results are based on model 3.

We investigated the performance of each summary statistic, and combinations of summary statistics, for estimating the divergence time *T*. We investigated the complex model 3 by simulating 49 "observed" datasets from a set of known parameter values using the same approach as described above. The ABC with the local linear regression adjustment was used to infer the population divergence time *T*. The mean difference between the true and the estimated *T*, and the mean width of the 95% credibility interval of the posterior sample of *T *is shown in Figure 7 and Additional file 1: Table S2 for each summary statistic, for pairs of summary statistics, and for the combination of all summary statistics. We first noted that an accurate mean of the posterior sample (small deviation from the true parameter-value) also corresponded to a narrow credible interval (Pearson correlation: 0.95, p < 10^-18^). Moreover, pairs of summary statistics generally improved the accuracy of the parameter estimation compared to single summary statistics; the mean difference between true and estimated *T *was greater than 0.070 for all single summary statistics (mean difference across the 8 summary statistics equaled 0.110), whereas the mean difference was less than 0.070 for 68% of the pairs of summary statistics (mean difference across the 26 pairs of summary statistics equaled 0.055). However, there were pairs of summary statistics that performed poorly - at the level of single summary statistics - for example, pairs that include *F_ST _*generally performed poorly, as well as pairs that included similar types of data, such as the number of distinct haplotype-alleles (NOA) and the number of private haplotype-alleles (NPA, Figure 7, Additional file 1: Table S2). The combination of all eight summary statistics estimated the divergence time *T *accurately (the mean difference was 0.0203 and the mean width of the 95%-credible interval was 0.1669), but several pairs of summary statistics performed at the same level. Although this comparison of summary statistics was by no means exhaustive, we noted that i) combining summary statistics generally provided more accurate inference, and ii) there was a large variation in performance across pairs of summary statistics. These two observations suggested that combining several summary statistics that capture different population-genetic phenomena may be a powerful approach for making accurate inferences at the same time as keeping the number of summary statistics low, both important features for any ABC investigation [4].

### Increasing the number of loci

**Figure 8:**
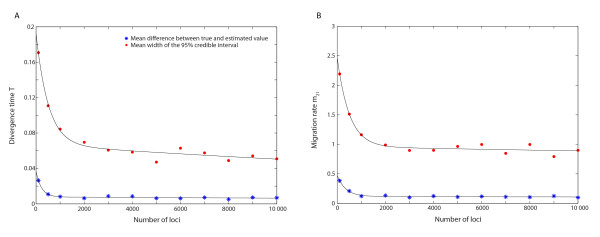
**The mean difference between the true and the estimated parameter value and the width of the 95% credible interval as functions of the number of loci for A) the divergence time *T *and B) the migration rate *m*_12_**.

We used a simulation approach to investigate the impact of the number of loci on accuracy of the ABC estimation, in particular the accuracy of the divergence time estimate and the migration rate estimates. We simulated 147 "observed" dataset from model 1 (each true parameter was varied for 49 values, *T *= 0.01, 0.02, ..., 0.49; *m*_12 _= 0.1, 0.2, ..., 4.9; and *m*_21 _= 0.1, 0.2, ..., 4.9, and the other true parameters were set as in Table 1) for increasing numbers of loci (100; 500; 1,000; 2,000; 3,000; 4,000; 5,000; 6,000; 7,000; 8,000; 9,000 and 10,000 genome-regions) and used the ABC approach to infer the divergence time *T *and migration rates. For increasing numbers of loci, Figure 8 shows the mean difference (across 49 choices of true parameter values for each parameter) of the true value and the mean of the posterior sample and the width of the 95% credible interval for the divergence time *T *and the migration rate *m*_21 _(the results for *m*_12 _were very similar to *m*_21_). The mean values of the posterior samples rapidly approach the true parameter value when the number of loci increases from 100 to 1,000 and the width of the 95% credible intervals rapidly decrease until about 2,000 loci, and continue to decrease at a low rate for increasing numbers of loci.

## Discussion

From the comparison of the three different models, it was clear that the accuracy in the parameter estimates was lower for the more complex model 3 with seven unknown parameters compared to models 1 and 2, which have three and four unknown parameters. This observation was not surprising since the less complex models were nested models within model 3. More generally, by increasing the number of simulated data sets in the ABC procedure, the parameter estimation was improved (especially the 95% credible interval). For example, for model 3, five of the seven parameter estimates were substantially improved if 50,000 replicates were used instead of 10,000 replicates, and the remaining two parameter estimates (the two current population sizes) were very similar regardless of the number of replicates. This result confirms that we can overcome some of the difficulties in parameter estimation for complex models by increasing the number of simulation steps in the ABC procedure, but it will mainly decrease the Monte Carlo error and allow a reduction of the tolerance level. However, the number of replicate simulations can be a limiting factor for ABC analyses of large scale genome-wide data because of computing time. In our case, approximately 8-9 minutes of CPU time was needed to generate one simulated dataset (10,000 genome regions of size 100 kb) for model 3 using *ms *[31], which corresponds to about 7,000 computer hours for 50,000 simulated datasets.

Among the seven parameters in our models, the migration rates and present population sizes were the most difficult to estimate. A divergence model without migration and an island migration model may result in quite similar gene genealogies for sampled individuals and there will be little information contained in most types of summary statistics to distinguish the differences [13]. Moreover, in a divergence model, the estimates of migration rates may depend on the divergence time since a model with large divergence time and large migration rates can generate gene genealogies that are similar to the gene genealogies of a model with short divergence time and small migration rates. However, under a scenario of divergence and gene flow, the variation in genealogical histories for different parts of the genome could in principle be used to separate migration rates and divergence times. To determine similarity between the "observed" data and the simulated data, we used the means of the summary statistics. Another option would be to use variance, quartiles (e.g. [39]), or a combination of different summaries of the distributions. We tested the performance of using the variances (instead of the means) of the summary statistics to infer the divergence time and the two migration rates in model 1 (true T = 0.1, *m*_12 _= 2.0, and *m*_21 _= 1.0). However, the precision of the parameter estimates were clearly more accurate based on means compared to using variances (0.1003 vs. 0.1008 for *T*, 1.9803 vs. 2.0915 for *m*_12_, and 0.9454 vs. 0.8234 for *m*_21_). If we use both the mean and the variance, the precision of the parameter estimates were quite close to the result based only on means, but with larger 95% credible intervals ([0.0952, 0.1063] vs. [0.0816 0.1218] for *T*, [1.6516, 2.1560] vs. [1.2458 3.0073] for *m_12_*, [0.5033, 1.3935] vs. [0.1575 1.6617] for *m_21_*). Note also that the number of summary statistics for the combined case is twice the number of summary statistics for the case based on means. The lower quality of the estimates may be related to increasing the dimensionality of the problem as the number of summary statistics increases when considering both means and variances [8].

In models 2 and 3, it is assumed that the population sizes grow exponentially and the populations spend a very short amount of time with a size close to the 'present population size', hence the populations are far from being at an equilibrium. The summary statistics that we used capture events over a long period of time, and during most of this time, the populations have much smaller sizes compared to the present population sizes. Therefore, little information about present population sizes was contained in the summary statistics, which could explain the difficulty in estimating present population sizes compared to past population sizes.

We investigated cases where the true parameter value was outside the range of the prior distribution (Table 6) in order to determine its effect on the parameter estimation and the potential warning signals to pay attention to. If we use the ABC approach with local linear regression adjustment including the transformation step, the posterior sample will be limited to the range of the prior distribution so the ABC practitioner needs to be observant of posterior distributions that are pushed close to the boundaries of the prior. For comparison, the ABC approach with local linear regression adjustment without a transformation step produce mean values of the posterior samples that were often fairly close to the true values despite that the range of the prior distribution did not overlap with the true value (but note that such results violate Bayes' theorem, see above). In many Bayesian analyses, when the prior distribution does not include the true value of the parameter in its support, the posterior sample might be skewed towards the true value indicating that something might be wrong with the choice of prior distribution. The ABC approach with local linear regression adjustment and transformation seem to preserve this property, which is reassuring. In practice, if the posterior distribution ends up close to a bound on the prior, we should adjust the range of prior distribution, so that the posterior is well within the prior distribution.

Huang *et al. *2011 [28] demonstrated increasing power for inferring divergence times with increasing numbers of loci, but limited their investigation to relatively small numbers of loci (< 100). We investigated much larger numbers of loci and found that the mean values of the posterior sample approach the true values when approx. 1,000 loci (or more) were used (Figure 8). The width of the 95% credible interval decreases rapidly as the number of loci increase from 100 to some 2,000, after which the decrease rate of the 95% width was lower. The same trends were observed for both the divergence time and migration rates (Figure 8). These results suggest that increasing the number of loci from around a hundred to several thousand improves the accuracy of parameters estimation using ABC. Although the greatest improvement appears for less than 2,000 loci, we note that model 1 is a relatively simple model, and for more complex models, the accuracy of the parameter estimation may continue to improve beyond 2,000 loci.

Both the mutation and the recombination rates are likely to vary across the genome. However, we assumed that the mutation and recombination rates did not vary along the genome, and they were fixed to a known value for the simulations used in the ABC. We further demonstrate that this assumption works well even if the mutation and the recombination rates vary around some mean values as long as these mean values are similar to the fixed values used in the ABC. To make the simulations in the ABC approach even more realistic, we could draw mutation and recombination rates for each genome-region from some distribution and potentially estimate the empirical mutation and recombination rates. Alternatively, the mutation and recombination rates could be treated as nuisance parameters that are only included to make the simulated data better resemble the empirical data.

## Conclusions

To conclude, we find that increasing the amount of data from a few loci, or a few hundred loci, to thousands of loci can substantially improve the accuracy of parameter estimation using ABC. In contrast to many full-likelihood inference approaches, the ABC approach is well suited for analyzing large amounts of population genomic data, using for example haplotype-based summary statistics.

## Authors' contributions

SL conducted the simulations and the analysis, MJ conceived and supervised the project, and SL and MJ wrote the manuscript. Both authors read and approved the final manuscript.

## Supplementary Material

Additional file 1 **Table S1**. Outline of the investigations of the performance of ABC using simulated datasets (called "observed" data) to mimic empirically observed data. **Table S2**. The mean difference between the true and estimated divergence time T (across 49 choices of true T) and the mean width of the 95% credible interval of the posterior sample given by single summary statistics, pairs of summary statistics, and the combination of all eight summary statistics. The results are based on model 3. See also Figure 7 in main text. Click here for file
