# Supplementary material for: Estimating demographic parameters from large-scale population genomic data using Approximate Bayesian Computation
Source: BMC Genet. 2012 Mar 27;13:22. doi: 10.1186/1471-2156-13-22 (PMC3368717; doi:10.1186/1471-2156-13-22)
Supplement: Additional file 1 — Table S1. Outline of the investigations of the performance of ABC using simulated datasets (called "observed" data) to mimic empirically observed data. Table S2. The mean difference between the true and estimated divergence time T (across 49 choices of true T) and the mean width of the 95% credible interval of the posterior sample given by single summary statistics, pairs of summary statistics, and the combination of all eight summary statistics. The results are based on model 3. See also Figure 7 in main text. [file 1471-2156-13-22-S1.DOC]

# Supplementary information for “Estimating demographic parameters from large-scale population genomic data using Approximate Bayesian Computation”

Sen Li and Mattias Jakobsson

**Supplementary Table S1**. Outline of the investigations of the performance of ABC using simulated datasets (called “observed” data) to mimic empirically observed data.

| **Model 1**  (98 datasets) | **Different divergence time *T*** | | **Different migration rate *m*_12_** | |
| --- | --- | --- | --- | --- |
|  | 49 “observed” datasets where *T* is set to 0.01, 0.02, ..., 0.49 for each dataset. | | 49 “observed” datasets where *m*_12_ is set to 0.1, 0.2, ..., 4.9 for each dataset. | |
|  | | | | |
| **Model 3**  (195 datasets) | **Different *T*** | **Different *m*_12_** | **Different past population size *N*_1_’** | **Different present population size *N*_1_** |
|  | 49 “observed” datasets where *T* is set to 0.01, 0.02, ..., 0.49 for each dataset. | 49 “observed” datasets where *m*_12_ is set to 0.1, 0.2, ..., 4.9 for each dataset. | 49 “observed” dataset where *N*_1_’ is set to [0.02, 0.04, ..., 0.98]×*N*_e_ for each dataset. | 48 “observed” datasets where *N*_1_ is set to [1.1, 1.5, ..., 19.9]×*N*_e_ for each dataset. |
|  | | | | |
| **Model 3**  (300 datasets) | **Variable mutation rate** | | **Variable recombination rate** | |
|  | 150 observed dataset with parameters set as in Table 1, but where the mutation rate of each locus was drawn randomly from a normal distribution with parameter [5/3, 0.2] (50 datasets), [5, 0.2] (50 datasets) , [15, 0.2] (50 datasets). The recombination rate was set to 40. | | 150 observed dataset with parameters set as in Table 1, but where the recombination rate of each locus was drawn randomly from a normal distribution with parameter [20, 0.2] (50 datasets), [40, 0.2] (50 datasets) , [80, 0.2] (50 datasets). The mutation rate was set to 5. | |

**Supplementary Table S2**. The mean difference between the true and estimated divergence time *T* (across 49 choices of true *T*) and the mean width of the 95% credible interval of the posterior sample given by single summary statistics, pairs of summary statistics, and the combination of all eight summary statistics. The results are based on model 3. See also figure 8 in main text.

| Summary statistic(s) | Mean diff. | Mean width of 95% interval |
| --- | --- | --- |
| FST | 0.1234 | 0.4385 |
| HAW | 0.1139 | 0.4499 |
| HHA | 0.1192 | 0.4620 |
| HSS | 0.0720 | 0.3338 |
| LDR | 0.1275 | 0.4324 |
| NOA | 0.1092 | 0.4608 |
| NPA | 0.1050 | 0.4555 |
| TAD | 0.1079 | 0.4141 |
| FST+HAW | 0.1171 | 0.4403 |
| FST+HHA | 0.1068 | 0.4121 |
| FST+HSS | 0.0479 | 0.2945 |
| FST+LDR | 0.1243 | 0.4142 |
| FST+NOA | 0.0974 | 0.3946 |
| FST+NPA | 0.1019 | 0.3995 |
| FST+TAD | 0.0820 | 0.3977 |
| HAW+HHA | 0.0408 | 0.1606 |
| HAW+HSS | 0.0133 | 0.1733 |
| HAW+LDR | 0.0212 | 0.2861 |
| HAW+NOA | 0.0384 | 0.1811 |
| HAW+NPA | 0.0501 | 0.2455 |
| HAW+TAD | 0.0143 | 0.1700 |
| HHA+HSS | 0.0173 | 0.1668 |
| HHA+LDR | 0.0226 | 0.1693 |
| HHA+NOA | 0.0691 | 0.3422 |
| HHA+NPA | 0.0813 | 0.3559 |
| HHA+TAD | 0.0199 | 0.1649 |
| HSS+LDR | 0.0680 | 0.3004 |
| HSS+NOA | 0.0205 | 0.1612 |
| HSS+NPA | 0.0240 | 0.1820 |
| HSS+TAD | 0.0199 | 0.2302 |
| LDR+NOA | 0.0289 | 0.1942 |
| LDR+NPA | 0.0287 | 0.1598 |
| LDR+TAD | 0.1168 | 0.3360 |
| NOA+NPA | 0.1089 | 0.4429 |
| NOA+TAD | 0.0233 | 0.1598 |
| NPA+TAD | 0.0273 | 0.1835 |
| ALL | 0.0203 | 0.1669 |

The following code was used to generate one simulated dataset for models 1, 2 and 3 (10,000 genome-regions). Several values are specified for each replicate simulation using the ‘tbs’ option (see *ms* manual), these values were the same for each replicate simulation.

Model 1:  ms 200 10000 -t 5 -r 40 100000 -I 2 100 100 -n 2 0.5 -m 1 2 tbs -m 2 1 tbs -ej tbs 2 1 -en tbs 1 1.5 -F 10

Model 2:  ms 200 10000 -t 5 -r 40 100000 -I 2 100 100 -n 1 tbs -n 2 tbs -g 1 tbs -g 2 tbs -eg 0.1 1 0.0 -eg 0.1 2 0.0 -ej 0.1 2 1 -en 0.1 1 tbs -F 10

Model 3:  ms 200 10000 -t 5 -r 40 100000 -I 2 100 100 -n 1 tbs -n 2 tbs -g 1 tbs -g 2 tbs -m 1 2 tbs -m 2 1 tbs -eg tbs 1 0 -eg tbs 2 0 -ej tbs 2 1 -en tbs 1 tbs -F 10
